# Supplementary material for: PPARG governs adipogenic differentiation and cell state plasticity in well-differentiated and dedifferentiated liposarcoma
Source: Sci Adv. 2026 Jul 31;12(31):eaea6516. doi: 10.1126/sciadv.aea6516 (PMC13426422; doi:10.1126/sciadv.aea6516)
Supplement: Supplementary file 1 — Figs. S1 to S7 [file sciadv.aea6516_sm.pdf]

Science Advances
AAAS

Supplementary Materials for

PPARG governs adipogenic differentiation and cell state plasticity in well-differentiated and dedifferentiated liposarcoma

Blake R. Wilde *et al.*

Corresponding author: Brian E. Kadera, bkadera@mednet.ucla.edu;
Heather R. Christofk, hchristofk@mednet.ucla.edu

Sci. Adv. **12**, eaea6516 (2026)
DOI: 10.1126/sciadv.aea6516

This PDF file includes:

Figs. S1 to S7

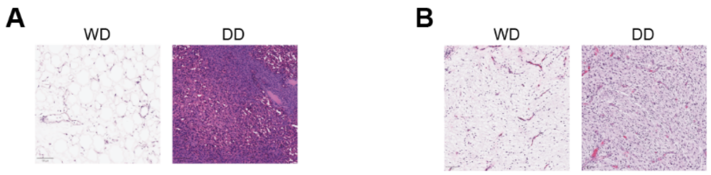

Fig. S1. Histologic characterization of WD and DD tumor components. (**A-B**) H&E staining of two WD/DD LPS tumors used in snRNAseq shows consistent histological differences between WD and DD regions, with WD components displaying abundant mature adipocytes and lipoblasts, and DD regions exhibiting dense cellularity and spindle morphology.

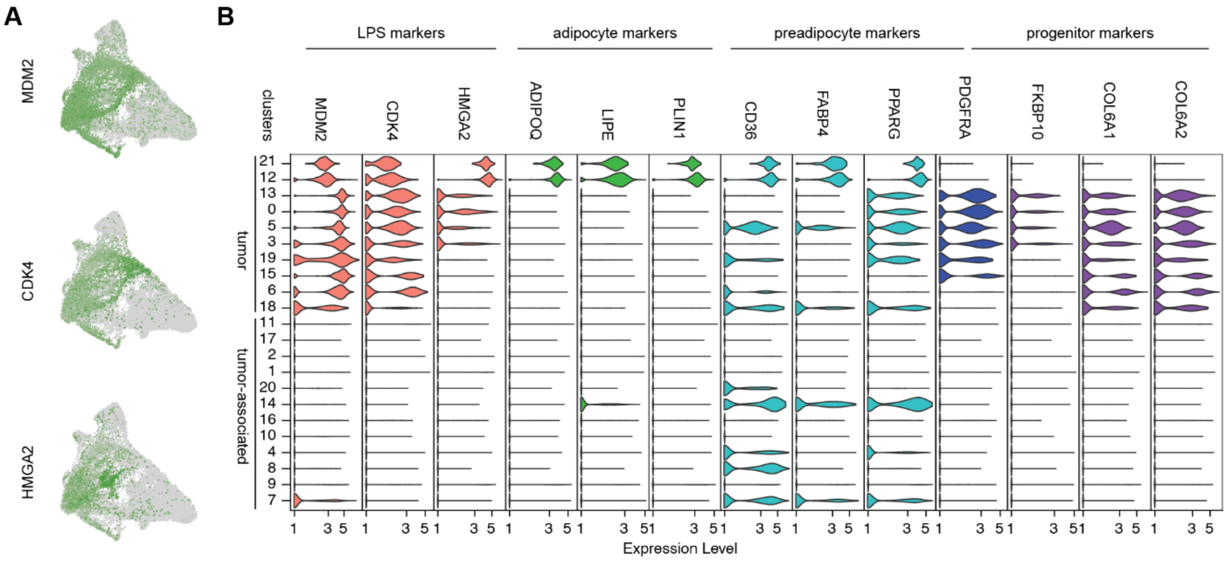

Fig. S2. Identification of tumor clusters based on expression of LPS oncogenes and copy number alterations. (**A**) UMAP feature plots for *MDM2*, *CDK4*, and *HMGA2* across all nuclei. (**B**) Violin plots further confirm selective expression of these oncogenes as well as adipocyte, preadipocyte, and adipocyte progenitor markers.

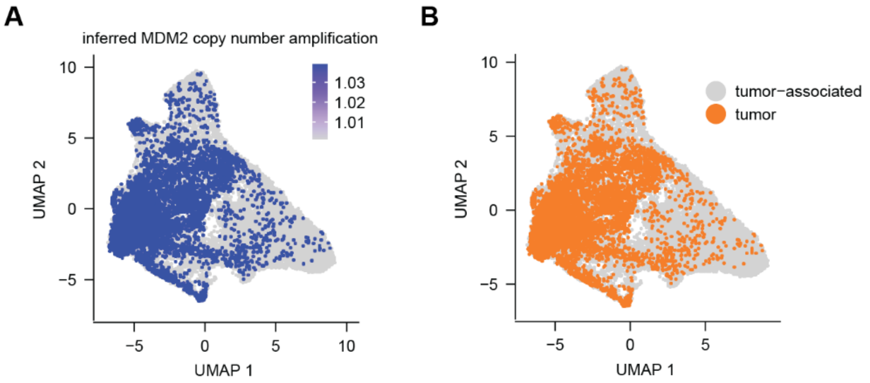

Fig. S3. Inferred copy number gains in MDM2 are used to identify tumor cells. UMAP profiles across all clusters highlight (**A**) amplification of MDM2 and (**B**) annotation of tumor cell populations.

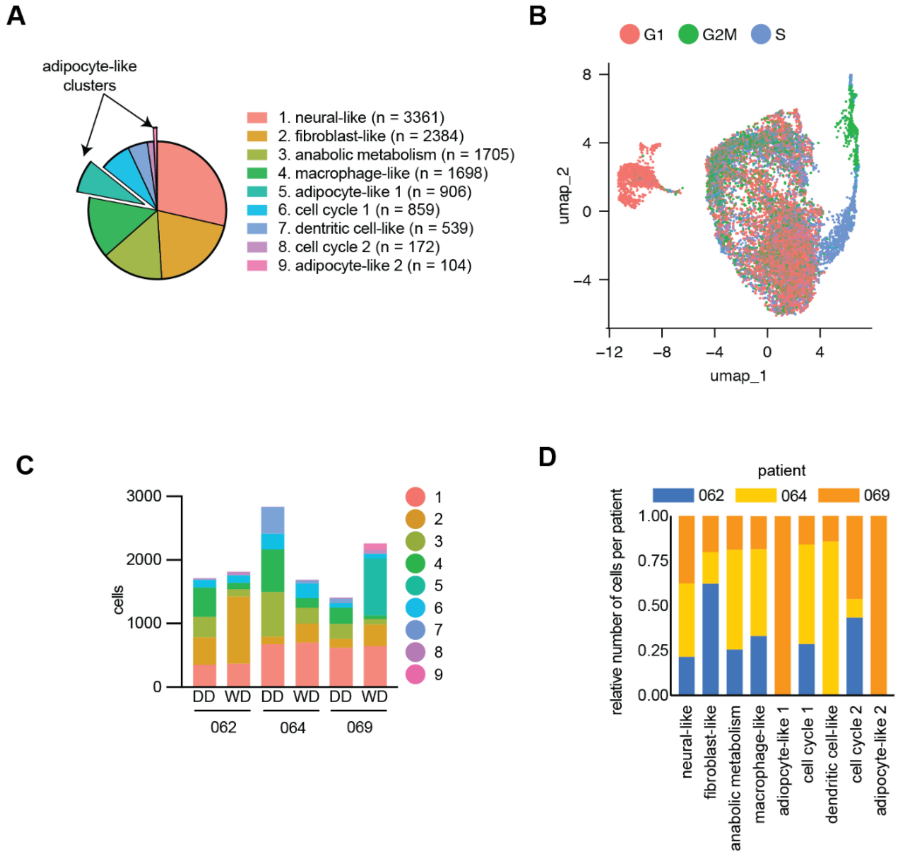

Fig S4. Functional annotation of re-clustered tumor cell states. (**A**) Pie chart shows relative number of nuclei across the nine malignant clusters, including corresponding to proliferative, metabolic, mesenchymal-like, immune-like, and adipocyte-like states. (**B**) UMAP plots colored by predicted cell cycle state of each nuclei. (**C**) Number of nuclei contributing to each cluster, split by patient and component. (**D**) The relative number of nuclei per patient for each cluster.

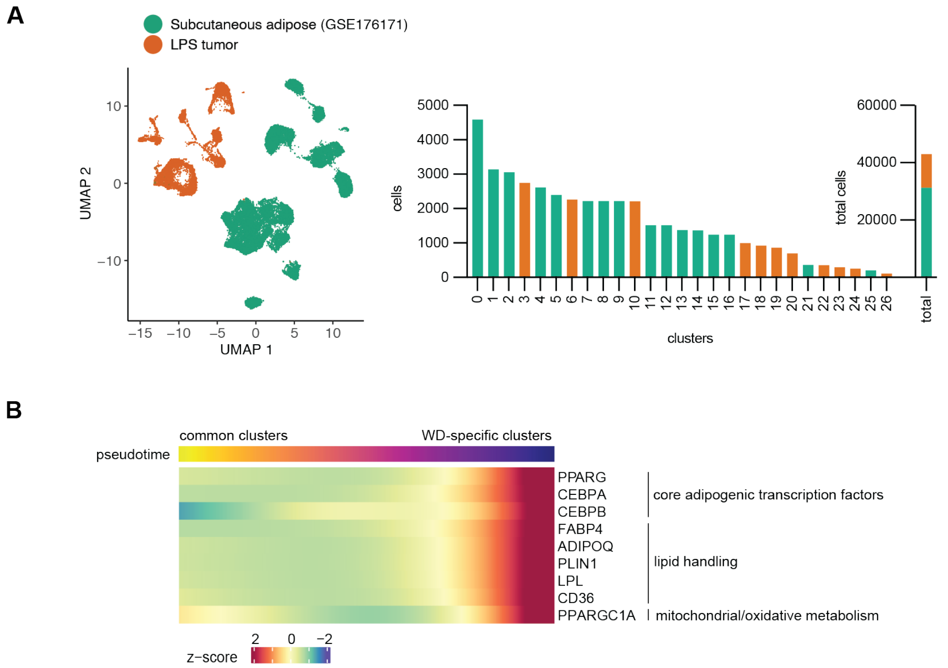

Figure S5. Comparison of tumor-associated adipogenic trajectories with normal adipocyte differentiation. (**A**) UMAP projection of WD/DD liposarcoma snRNA-seq data integrated with a published single-cell RNA-seq dataset of human adipose tissue spanning adipose stem and progenitor cells to mature adipocytes (GEO accession #GSE176171). Tumor cells and normal adipose cells occupy largely non-overlapping regions, indicating transcriptional distinction between LPS adipocyte-like cells and physiologic adipocytes. (**B**) Expression of canonical adipogenic gene modules along the WD pseudotime trajectory, demonstrating progressive induction of PPARG target genes, lipid-handling enzymes, and mitochondrial metabolism programs similar to those engaged during normal adipogenesis.

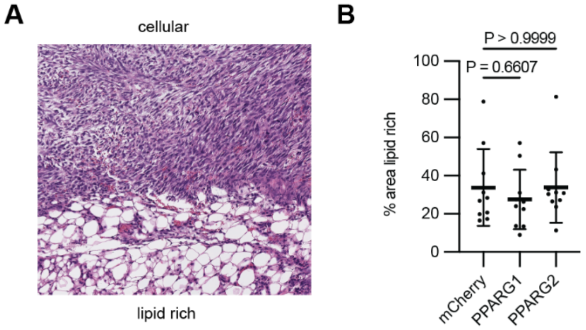

Fig S6. Histological analysis of LPS2 xenografts with induction of PPARG isoforms. (**A**) Representative H&E staining of LPS2 xenograft tumors following doxycycline-induced expression of PPARG1 or PPARG2 reveal focal lipid-rich regions. (**B**) Quantification of lipid-positive area shows no differences, likely due to limited tumor size following PPARG-mediated growth suppression.

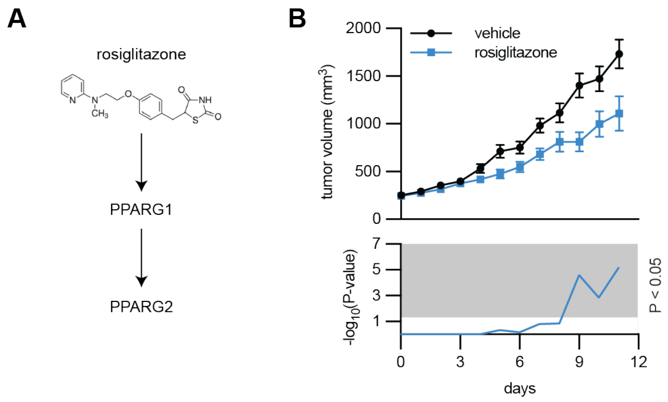

Fig S7. PPARG activation decreases DD LPS tumor growth in vivo. (**A**) Rosiglitazone activates PPARG1 activity, which drives expression of PPARG2. (**B**) Rosiglitazone treatment of mice bearing parental LPS2 xenografts reduces tumor growth, supporting the idea that pharmacologic activation of PPARG1 can partially recapitulate the effects of PPARG2 *in vivo*.
